# Effects of mind-body exercise on PTSD symptoms, depression and anxiety in PTSD patients: A protocol of systematic review and meta-analysis

**DOI:** 10.1097/MD.0000000000024447

**Published:** 2021-01-29

**Authors:** Lin Zhu, Long Li, Xiao-zhi Li, Lin Wang

**Affiliations:** a School of Physical Education, Soochow University, Suzhou; b Department of Physical Education, Southeast University, Nanjing, Jiangsu; c Department of Physical Education, Wuhan University of Technology, Wuhan, China.

**Keywords:** anxiety, depression, meta-analysis, mind-body exercise, PTSD symptom, systematic review

## Abstract

**Background:**

The present study aimed to systematically analyze the effects of mind-body exercise on PTSD symptom, depression and anxiety among patients with post-traumatic stress disorder (PTSD) and to provide a scientific evidence-based exercise prescription. Meanwhile, it will also help reduce the global mental health burden of COVID-19.

**Methods:**

Both Chinese and English databases (PubMed, Web of Science, the Cochrane Library, EMBASE, VIP Database for Chinese Technical Periodicals, China National Knowledge Infrastructure, and Wanfang) were used as sources of data to search for randomized controlled trials (RCTs) published between January 1980 to September 2020 relating to the effects of mind-body exercise on PTSD symptom, depression and anxiety in PTSD patients.

**Conclusion:**

This systematic review and meta-analysis will provide stronger evidence on the effectiveness and safety of mind-body exercise for PTSD symptoms in PTSD patients.

**Systematic review registration:**

INPLASY2020120072.

## 1 Introduction

The coronavirus disease 2019 (COVID-19) pandemic has precipitated a global mental health crisis. Internationally, the onset of COVID-19 has been linked to increased anxiety, depression, substance use, stress response, and suicidal ideation in the general population.^[1]^ The impact of PTSD is multi-faceted. According to the DSM-5, the diagnosis of PTSD is characterized by 4 broad symptom clusters that include intense reliving of the traumatic event through disruptive memories and nightmares, avoidance of reminders of the event, negative cognitions and mood, and hyperarousal.^[2]^ In addition to the characteristic symptoms of PTSD, impaired cognitive performance^[3,4]^ and alterations in brain structure and function^[5,6]^ are well-documented. Individuals with PTSD suffer substantial social and interpersonal problems, as well as impaired quality of life stemming from the long-term presence of the intrusive, avoidant and hyperaroused symptoms that characterize the disease. Although conventional pharmacologic and psychotherapeutic interventions have shown some proven efficacy in the treatment of PTSD,^[7]^ residual symptoms and therapeutic efficacy remain problematic. In recent years, complementary therapy approaches for individuals with PTSD and other trauma-related disorders have received increasing interest.^[8]^

Mind-body exercise, as a way to promote physical and psychological health, has received recent attention in the scientific literature. Mind-body exercise focuses on mind, body, psychology, and behavior, including breathing and physical exercise, meditation, and so on.^[9,10]^ Emphasizing trinity of mind, body, and breathing,^[11,12]^ it has the advantages of physical and psychological exercise.^[13–15]^ Recently, a variety of integrative mind-body intervention modalities have emerged that are increasingly employed in the treatment of PTSD. In 2010, 39 percent of individuals with PTSD reported using complementary and alternative medicine (CAM) interventions, including mind-body practices that incorporate various types of stretching movements and postures combined with deep breathing (e.g., yoga, tai chi, qigong, and meditation).^[16]^ Indeed, many studies have shown that mind-body exercise has the potential to exert a positive impact on PTSD via both psychological and neurophysiological mechanisms, such as exposure and desensitization to internal arousal cues, enhanced cognitive function, exercise-induced neuroplasticity, normalization of hypothalamic pituitary axis (HPA) function, and reductions in inflammatory markers.^[17–19]^ Furthermore, there is emerging evidence that supports the neural and biological mechanisms underlying mind-body practices for the management of stress related illness.^[20–22]^ Thus, mind-body exercise potentially could have a positive impact on PTSD, depression, and anxiety symptoms and may have high utility as an adjunctive/complementary treatment. Meanwhile, it will also help reduce the global mental health burden of COVID-19.

## 2 Methods

This review protocol is registered in the International Platform of Registered Systematic Review and Meta-analysis Protocols, registration number INPLASY2020120072. Cochrane Handbook for Systematic Reviews of Interventions will be used as guidance to conduct this systematic review. Meanwhile, the “preferred reporting items for systematic reviews and meta-analyses” (PRISMA) statement^[23]^ will serve as guidelines for reporting present review protocol and subsequent formal paper. Ethical approval will not be necessary since this systematic review and meta-analysis will not contain any private information of participants or violate their human rights.

### 2.1 Inclusion criteria for study selection

#### 2.1.1 Types of studies

The inclusion criteria were as follows:

1. randomized controlled trial (RCT),
2. studies reported in full-text will be screened for inclusion,
3. outcome indicators including test data on PTSD, depression, and anxiety; and
4. publication language of Chinese or English.

#### 2.1.2 Types of participants

We will include trials of adult (18 years of age or older) human, psychiatrist-confirmed DSM-IVTR diagnosis of PTSD; medical clearance to participate in an exercise programme; and cognitively able to provide consent to participate. We will exclude studies of people reported substance dependence, psychosis, or use of alphaor beta-blocking medications because of possible interference with psychophysiological measures.

#### 2.1.3 Types of interventions

Our systematic review and meta-analysis will be conducted based on the RCTs that mind-body exercise in the experimental group and regular daily life in the control group. The experimental group included mindfulness, yoga, taichi, qigong, meditative movement, etc. The control group included usual care, no physical activity, and no-intervention control group and other different types.

#### 2.1.4 Types of outcome measurements

The primary outcome was PTSD symptoms as assessed by the PTSD check-List-civilian version (PCL-C).^[24]^ The PCL-C is a 17-item self-report questionnaire that assesses the key symptoms of PTSD and is one of the most commonly used self-report measures of PTSD.^[25]^ Scores range from 17 to 85, with higher scores indicating higher symptom severity and a score of 45 normally selected as the cutoff for a diagnosis of primary PTSD.^[24]^

Secondary outcomes. The Depression Anxiety and Stress Scale (DASS) is a 42-item self-report instrument that measures the related negative emotional states of depression and anxiety. Higher scores on the DASS equate to greater symptom severity. For the depression domain, scores of 0–13 are considered normal or mild, 14–20 moderate, 21–27 severe, and >28 extremely severe. For anxiety, 0–7 is considered normal, 8–9 mild, 10–14 moderate, 15–19 severe, and >20 extremely severe.^[26]^ The psychometric properties of the DASS have been comprehensively evaluated, and it has been found to be valid, consistent, and responsive to treatment.^[26]^

### 2.2 Search strategy

We will systematically search the following databases: PubMed, Web of Science, the Cochrane Library, EMBASE and VIP Database for Chinese Technical Periodicals, China National Knowledge Infrastructure, and Wanfang. These databases will be search to identify randomized controlled trials (RCTs) published in any language between January 1, 1980, and September 30, 2020. The search terms will include “mindfulness” or “mind-body exercise”, and “yoga” or “taichi” or “qigong” or “meditation”, with PTSD terms including “COVID-19, PTSD, Post-traumatic stress disorder”, as well as, “depression, anxiety, depressive disorder, anxiety disorder”.

### 2.3 Data collection and analysis

#### 2.3.1 Selection of studies

**Figure 1:**
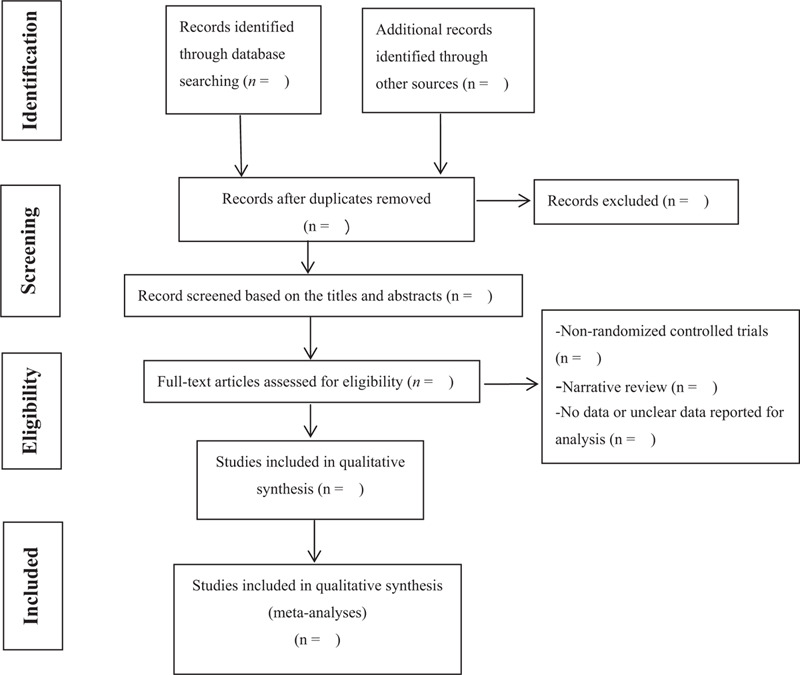
Flow diagram of study selection. From: Moher D, Liberati A, Tetzlaff J, Altman DG, The PRISMA Group (2009). Preferred Reporting Items for Systematic Reviews and Meta-Analyses: The PRISMA Statement. PLoS Med 6(7): e1000097. doi:10.1371/journal.pmed1000097. For more information, please visit: www.prisma-statement.org.

Two investigators (Lin Zhu and Lin Wang) independently reviewed the titles and abstracts from the search results and screened the full texts of references that might be eligible. If a study met eligibility requirements, it received a full-text article assessment. When any disagreement between the 2 review authors occurred, a third review author (Long Li) was invited to verify the eligibility of the uncertain article by discussion with them. All eligible studies included information such as author, publication year, country, age, sample size, intervention methods, duration, measurement standards, results, and dropouts. Details of the entire selection procedure will be shown in a PRISMA flow chart^[27]^ (Fig. 1).

#### 2.3.2 Data extraction and management

We will make a detailed data and information extraction form consisted mainly of following items:

1. Publication information (Name of first author, Year, Country);
2. Participants characteristics (Sample size, Age, Sex ratio, intervention methods, Duration, Measurement standards, Follow-up);
3. Interventions (Exercise type, Frequency, Duration and duration of each intervention session);
4. Comparison (Treatment types, Frequencies, Treatment time, Course of treatment);
5. Outcomes and others (Scale instruments, Outcome assessments, Informed consent, Drop-out rate and reasons,);
6. Study design (Randomization, Blinding).

All above information or data will be obtained through reading the full text.

#### 2.3.3 Appraisal of study quality

Two authors will use the modified the Physical Therapy Evidence Database (PEDro) scale [39] to independently perform methodological quality assessment of each eligible study. This assessment will consist of 9 items (randomization, concealed allocation, similar baseline, blinding of assessors, = <15% dropouts, intention-to-treat analysis, between-group comparison, point measure and measures of variability, isolate exercise intervention), and higher scores indicate better quality of the method.

#### 2.3.4 Assessment of reporting bias

We will generate funnel plots to assess reporting bias. For continuous variables, Egger test will also be taken to test funnel plot asymmetry. Asymmetric funnel chart is usually considered as a type of reporting deviation, but it also means that there may be other reasons, such as differences in methods or intervention cycle. We will analyze the possible reasons for the deviation and make a reasonable explanation for the asymmetric funnel diagram.

#### 2.3.5 Assessment of heterogeneity

We will assess the heterogeneity applying the Chi-Squared and *I*^2^ test. *I*^2^ values of 25%, 50%, and 75% are considered as low, moderate, and high heterogeneity, respectively.^[28]^ We will analyze the possible sources of high heterogeneity when it emerges.

#### 2.3.6 Measure of treatment effect

According to the Cochrane Collaboration handbook for systematic reviews of interventions, selection of fixed- or random-effects meta-analysis should be based on the potential real effect of an intervention on outcome measures. Differences (standard mean difference, SMD) and 95% confidence intervals (95% CIs) were calculated. SMD was considered as small (0.2–0.49), moderate (0.5–0.79), or large (0.8).

#### 2.3.7 Data synthesis

Stata 14.0 will use to carry out the meta-analysis: mapping overall forest plot, heterogeneity analysis, regression analysis, and sub-group analysis. When the heterogeneity test *I*^2^ ≥ 50%, a random-effects model will be use for meta-analysis. Otherwise, the fix-effect model will be adopted.

#### 2.3.8 Subgroup analysis

Subgroup analyses will be conducted which aims to explain the potential causes of heterogeneity when necessitated. The subgroup analyses will be implemented according to the age, gender, frequency, time, duration, and even.

#### 2.3.9 Sensitivity analysis

After the data is synthesized, we will conduct sensitivity analysis by excluding the combined studies one by one to observe whether the synthesized results have changed significantly. If there is a significant change indicating that the exclusion of the study has a significant impact on the results, it should be reassessed. If there is no significant impact, the comprehensive data result is reliable.

#### 2.3.10 Quality of evidence

Two authors (Zhu and Wang) will use the modified the Physical Therapy Evidence Database (PEDro) scale to independently perform methodological quality assessment of each eligible study. This assessment will consist of 9 items, and higher scores indicate better quality of the method. It should be noted that the use of this scale is highly recommended to assess the quality of trials for systematic reviews due to its reliability and validity.

## 3 Discussion

Recently, a variety of integrative mind-body intervention modalities have emerged that are increasingly employed in the treatment of PTSD. This growing body of evidence has shown that mind-body interventions have a positive impact on quality of life, stress reduction, and improvement of health outcomes among individuals with PTSD.^[23–28]^ Recently, a large number of studies have been carried out to evaluate the influence of mind-body exercise on PTSD symptoms, depression and anxiety among PTSD patients. Because of the differences in the intervention samples, timing, frequency, method, and duration, the specific effects on PTSD symptoms, depression and anxiety among PTSD patients could have been different. Therefore, the purposes of our meta-analysis will evaluate the effect of mind-body exercise on PTSD symptoms, depression and anxiety in PTSD patients. Meanwhile, this study will also explored the internal regulation mechanism of mind-body exercise on the PTSD symptoms, depression and anxiety of PTSD patients to provide a corresponding exercise prescription. At the same time, it can also provide exercise prescriptions to relieve traumatic stress disorder for patients around the world affected by COVID-19.

## Author contributions

Lin Wang and Lin Zhu contributed to the conception and design of the review; Lin Zhu and Xiao-zhi Li applied the search strategy and performed the data analysis; Long Li conducted methodological supervision; Lin Zhu wrote this manuscript. All authors have read and agreed to the published version of the manuscript.

**Conceptualization:** Lin Zhu, Long Li, Lin Wang.

**Data curation:** Lin Zhu.

**Formal analysis:** Long Li, Xiao-zhi Li.

**Funding acquisition:** Lin Wang.

**Investigation:** Lin Wang.

**Methodology:** Lin Zhu, Xiao-zhi Li.

**Project administration:** Long Li, Lin Wang.

**Resources:** Lin Zhu, Xiao-zhi Li.

**Software:** Lin Zhu, Lin Wang.

**Supervision:** Long Li.

**Validation:** Long Li, Xiao-zhi Li.

**Writing – original draft:** Lin Zhu.

**Writing – review & editing:** Long Li, Lin Wang.

## Abbreviations

Abbreviations: BDI-II = Beck Depression Inventory-II, BMT = brief mindfulness training, CAPS = Clinician Administered PTSD Scale, CES-D = Center for Epidemiologic Studies-Depression Scale, DASS 21 = Depression Anxiety and Stress Scale, DASS-42 = Depression Anxiety and Stress Scale, GDA = General Distress-Anxiety, GDD = General Distress-Depressive, HAM-D/-A = Hamilton Depression/Anxiety Rating scales, ISI = Insomnia Severity Index, MASQ = Mood and Anxiety Symptoms Questionnaire, MBSR = mindfulness-based stress reduction, MBX = mindfulness-based stretching and deep breathing exercise, PCL = PTSD Checklist, PSS = Perceived Stress Scale, RSQ = Relationship Structures Questionnaire, SCL-90-R = symptom checklist-90-revision, STAI = State Trait Anxiety Inventory, TAU = treatment as usual, W = week.
